# A rare cause of recurrent vomiting: large polypoid duodenal gastric heterotopia. A case report with a focused review of the paediatric literature

**DOI:** 10.3389/fped.2026.1931161

**Published:** 2026-09-30

**Authors:** Martina Begucci, Federica Natalizio, Renato Tambucci, Nicoletta Sgarro, Andrea Marzullo, Alberto Gaeta, Ruggiero Francavilla, Luigi Dall'Oglio, Fernanda Cristofori

**Affiliations:** 1 Postgraduate School of Pediatrics, University of Bari Aldo Moro, Bari, Italy; 2 Pediatric Hospital Bambino Gesù IRCCS, Gastroenterology and Nutrition Unit, Rome, Italy; 3 University of Bari Aldo Moro, Interdisciplinary Department of Medicine-Pediatric Section, Bari, Italy; 4 Pediatric Hospital Giovanni XXIII- Department of Radiology, Bari, Italy

**Keywords:** case report, duodenum, endoscopic therapy, gastric heterotopia, vomiting

## Abstract

Gastric heterotopia (GH) is a developmental anomaly characterized by the presence of ectopic gastric mucosa outside its normal location. It is rare in the duodenum, particularly as a large polypoid lesion in children. We report the case of a 12-year-old boy who developed recurrent postprandial nausea and vomiting at 11 years of age. Imaging and endoscopy revealed a large partially obstructing polypoid mass in the distal duodenum. Technetium-99m pertechnetate scintigraphy demonstrated ectopic gastric mucosa. The lesion was successfully removed through a two-stage endoscopic resection, leading to complete symptom resolution. Histopathological examination confirmed a hyperplastic polyp arising from heterotopic gastric mucosa in the distal duodenum. This case highlights that polypoid duodenal GH should be considered in children presenting with chronic unexplained vomiting or symptoms of partial upper intestinal obstruction. Complete resection resulted in symptom resolution in our patient and may be achieved endoscopically in selected cases.

## Introduction

Heterotopia refers to the presence of morphologically normal, mature tissue located in an abnormal anatomical site (1). In the gastrointestinal tract, gastric heterotopia (GH) is a developmental anomaly most commonly identified in the oesophagus, where it presents as an “inlet patch,” or within Meckel's diverticulum and intestinal duplications. Less frequent locations include the gallbladder, rectum, and various segments of the small intestine, such as the duodenum and jejunum (2).

Duodenal GH is most often detected incidentally during endoscopy, typically appearing as small nodular lesions confined to the duodenal bulb. In contrast, large polypoid lesions arising in the distal duodenum or jejunum are uncommon and have only rarely been reported.

When symptomatic, GH may manifest either through acid-related mucosal injury, due to ectopic oxyntic tissue, or through a mass effect.

Here, we report a rare case of a large, symptomatic polypoid GH located in the distal duodenum of a paediatric patient, presenting with vomiting. This case highlights the diagnostic challenges and supports the role of minimally invasive endoscopic management in selected patients and is complemented by a focused review of previously reported paediatric cases of GH involving the small intestine.

## Case presentation

A 12-year-old boy was referred to our unit for evaluation of recurrent postprandial vomiting. His previous medical and surgical history was unremarkable, except for regurgitation and occasional episodes of food-content vomiting during the first year of life, followed by a prolonged symptom-free period. At 11 years of age, he developed recurrent episodes of nausea and postprandial vomiting, predominantly triggered by large or rapidly consumed meals, with progressive worsening in frequency and impact on daily activities. Occasional episodes of bilious vomiting were also reported. No other gastrointestinal, respiratory, or cardiac symptoms were present. Growth was normal, with both weight and height at the 75th percentile and no weight loss. Physical examination, vital signs, and routine laboratory investigations were unremarkable.

Abdominal computed tomography (CT) revealed a 60 × 23 × 21 mm intraluminal mass in the distal duodenum, immediately proximal to the ligament of Treitz, causing partial luminal obstruction without upstream bowel dilatation and without evidence of infiltration of adjacent structures (Figure 1). Subsequent esophagogastroduodenoscopy (EGD) confirmed a large polypoid lesion with a short peduncle arising from the distal duodenum, causing partial luminal obstruction. The lesion was covered by macroscopically normal mucosa, without ulceration or active bleeding. The stomach and the remaining examined duodenum were unremarkable (Figure 2). Endoscopic biopsies raised suspicion of gastric heterotopia; however, the limited amount of tissue precluded a definitive histological diagnosis.

**Figure 1:**
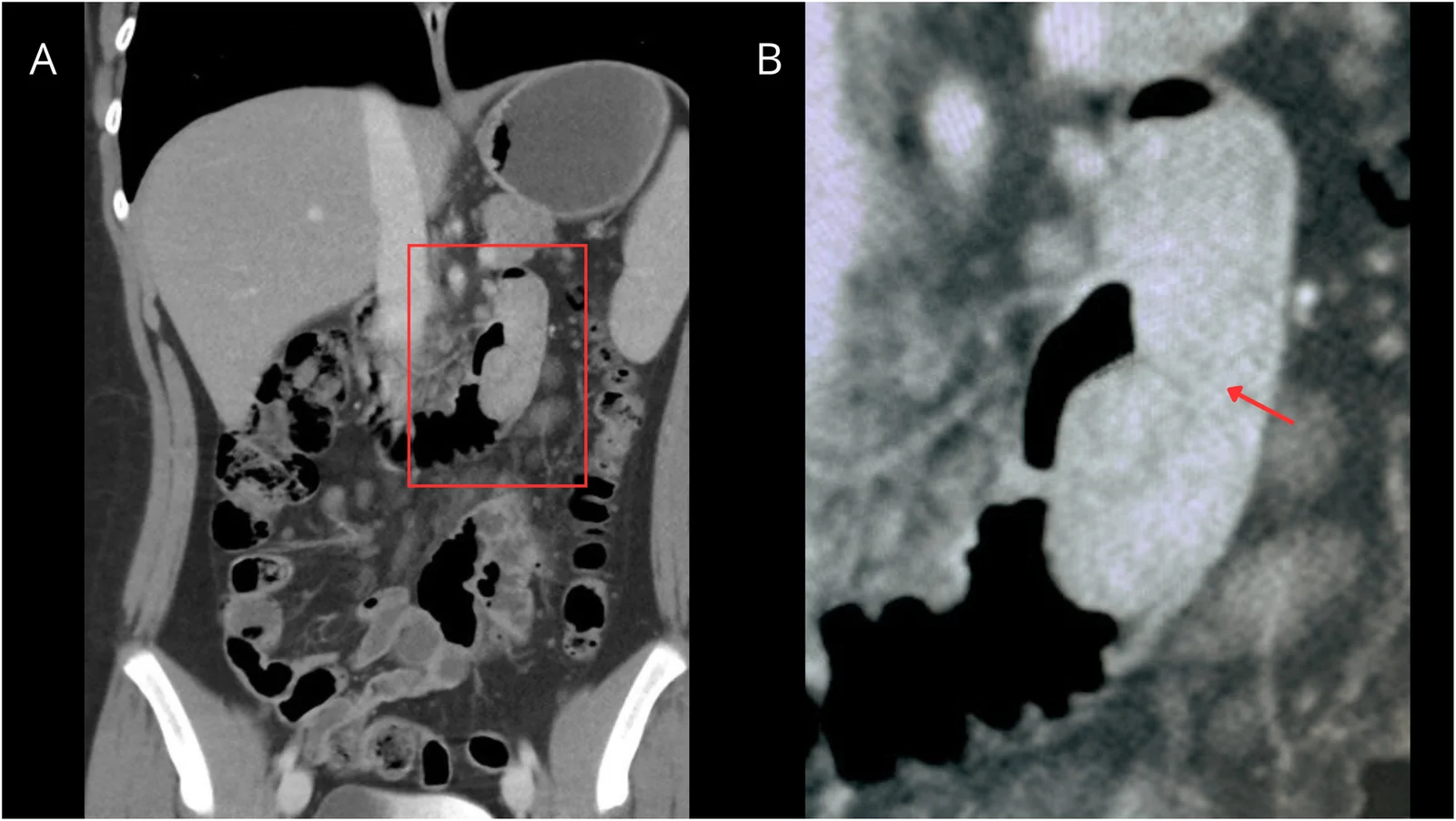
Contrast-enhanced coronal CT images demonstrating a 60 × 23 × 21 mm intraluminal mass in the distal duodenum, immediately proximal to the ligament of treitz. **(A)** Overview image showing the anatomical location of the lesion (red box). **(B)** Magnified view showing the lesion causing partial luminal obstruction without upstream bowel dilatation (red arrow).

**Figure 2:**
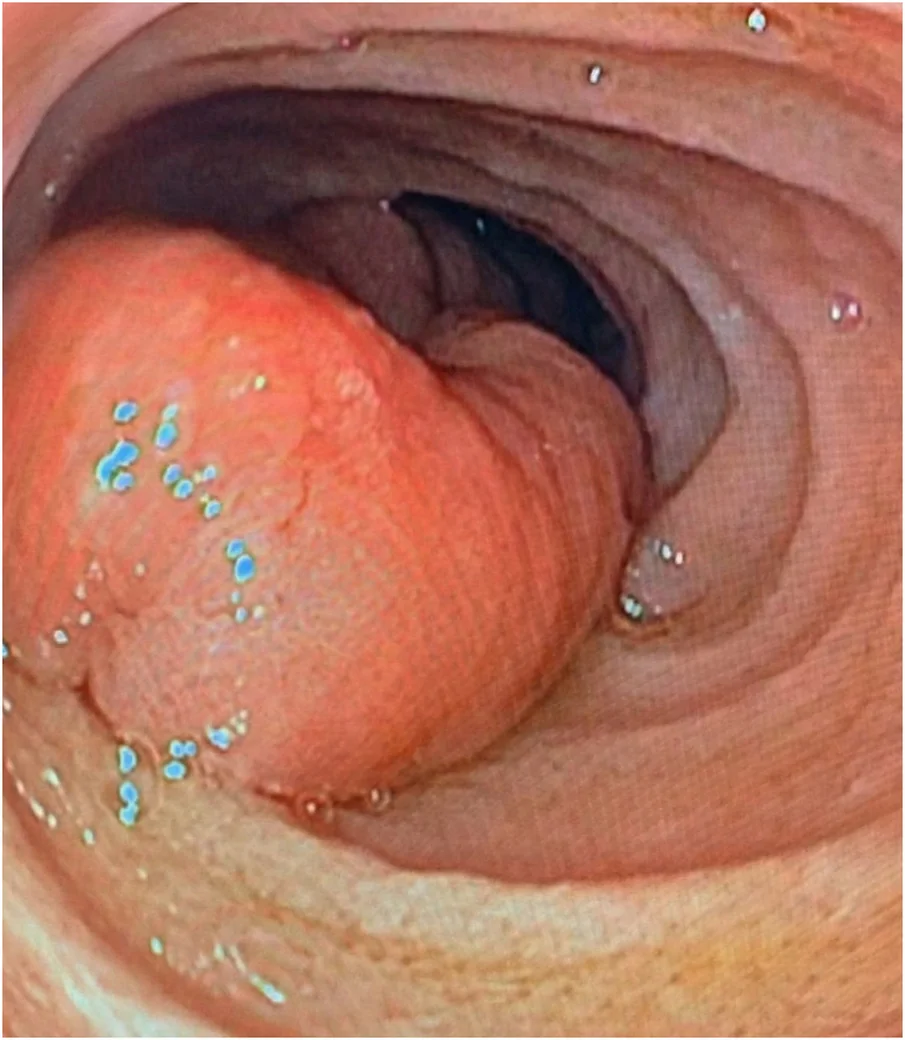
Endoscopic view of a large polypoid lesion with a short peduncle in the distal duodenum, immediately proximal to the ligament of Treitz, causing partial luminal obstruction. The lesion is covered by macroscopically normal mucosa, without ulceration or active bleeding.

Technetium-99m pertechnetate scintigraphy subsequently demonstrated ectopic gastric mucosa in the distal duodenum. No nasogastric decompression or specific pharmacological treatment was required before endoscopic treatment.

The lesion was successfully removed by piecemeal snare resection through a two-stage endoscopic approach under general anaesthesia. Complete resolution of vomiting was observed within 48 h after the first endoscopic procedure. The postoperative course was uneventful, with no procedure-related complications, and the patient was discharged home on postoperative day 5 in good clinical condition. Histopathological examination confirmed a hyperplastic gastric polyp arising from heterotopic gastric mucosa (Figure 3) with complete excision of the lesion. Follow-up endoscopy performed at 12 months showed no evidence of residual or recurrent lesion. The patient remained asymptomatic throughout 24 months of follow-up, with no recurrence of vomiting. A summary of the clinical course, including the key diagnostic and therapeutic steps, is provided in Table 1.

**Figure 3:**
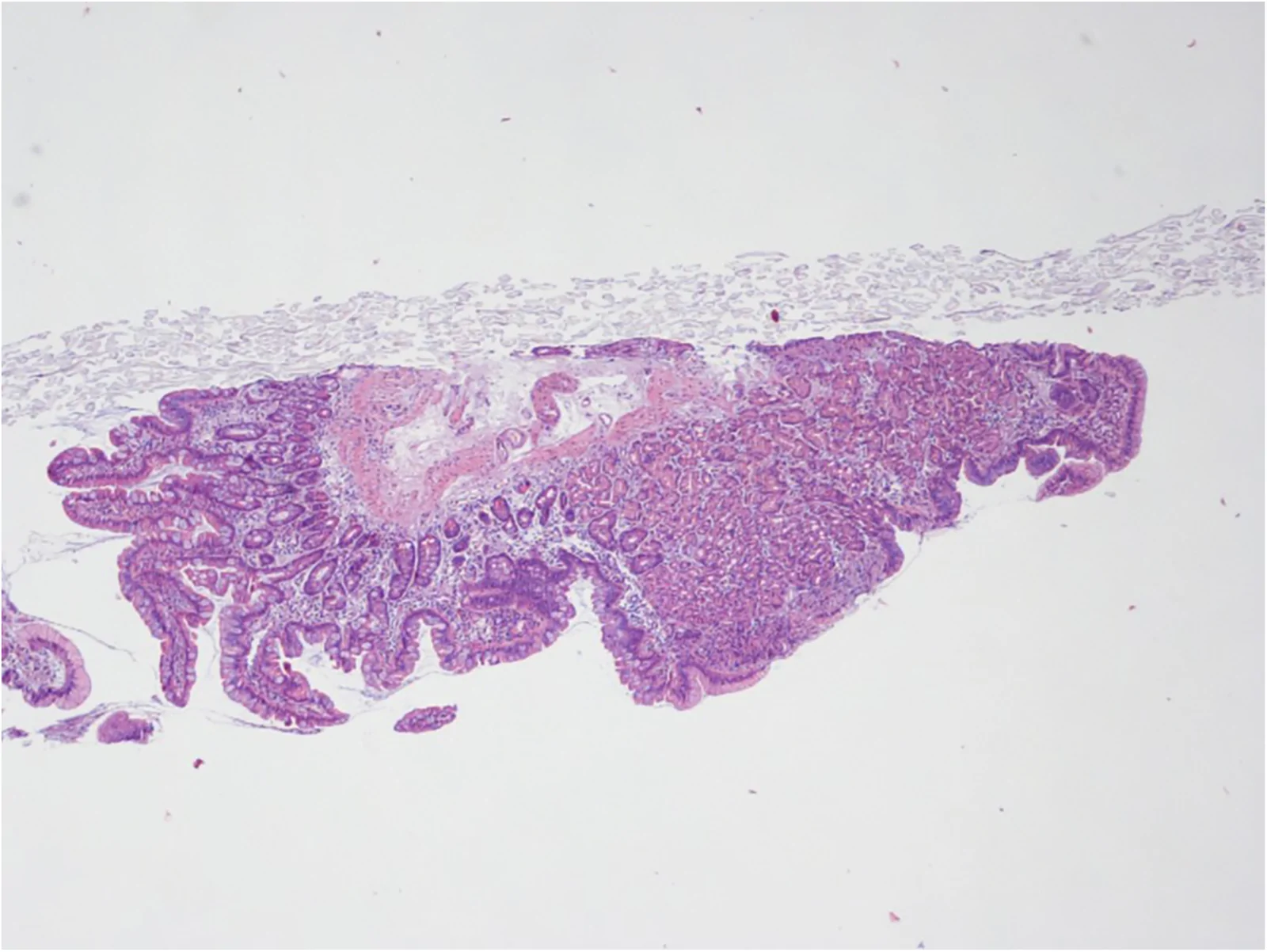
Histological section showing gastric-type mucosa adjacent to duodenal mucosa with preserved villous architecture. Haematoxylin and eosin staining; original magnification ×4.

**Table 1:** Timeline.

Timeline
1. Physical examination and routine laboratory tests in a 12-year-old boy with recurrent postprandial vomiting.
2. Hospital admission because of progressive worsening of postprandial vomiting
3. Abdominal computed tomography (CT) revealed a partially obstructing duodenal mass.
4. Esophagogastroduodenoscopy (EGD) confirmed a large polypoid lesion in the distal duodenum.
5. Technetium-99m pertechnetate scintigraphy demonstrated the presence of ectopic gastric mucosa in the duodenum.
6. Resection by piecemeal snare technique, a two-stage endoscopic approach, with complete resolution of vomiting.
7. Histopathological examination confirmed a hyperplastic gastric polyp arising from heterotopic gastric mucosa.

## Discussion

Gastric heterotopia (GH) of the small bowel represents a diagnostic challenge due to its rarity and heterogeneous clinical presentation (1). While ectopic gastric mucosa is most commonly identified within Meckel's diverticulum, isolated polypoid GH arising in the distal duodenum or at the duodenojejunal junction is rarely reported in the paediatric population, with the available evidence largely limited to individual case reports and small case series.

The distinction between gastric heterotopia and gastric metaplasia is clinically relevant. While metaplasia represents an acquired adaptive response to mucosal injury, heterotopia is a congenital condition that may occur throughout the gastrointestinal tract, from the oral cavity to the distal rectum (1, 2).

To better contextualize our findings, we performed a focused review of the literature on paediatric GH involving the small intestine. A structured search of PubMed/MEDLINE and Scopus databases was conducted up to 31 July 2026 using combinations of the terms “gastric heterotopia,” “heterotopic gastric mucosa,” “duodenum,” “small intestine,” and “paediatric.” The initial search was intentionally broad to identify potentially relevant small-bowel cases. Reports associated with Meckel's diverticulum were excluded, while paediatric cases of GH involving the duodenum or other small-bowel sites were eligible for inclusion. Overall, 314 records were identified; after removal of duplicates and initial screening, 63 articles concerning small-intestinal GH were assessed, of which six articles reporting 16 paediatric cases fulfilled the inclusion criteria and were included in Table 2 (3–8).

**Table 2:** Summary of reported paediatric cases of gastric heterotopia involving the small intestine, including demographic characteristics, clinical presentation, diagnostic findings, treatment, and complications.

Author	Year	Country	Age	Nr of cases	Sex	Symptoms	Diagnosis and Treatment	Localization	Complications
Begucci M. et al.	2026	Italy	12y	1	M	Recurrent episodes of nausea and vomiting	Abdominal SPECT/CT scan, 99mTc-pertechnetate scintigraphy, Two-stage endoscopic resection	Distal duodenum	None
Lambert M. P. et al. (3)	2000	USA	7y	1	M	Abdominal pain, vomiting, acute abdomen, massive gastrointestinal hemorrhage	Duodeno-duodenostomy, CT scan Autoptic diagnosis	Small intestine	Sepsis, death
Huang Q. et al. (4)	2025	China	4y5m	1	M	Anaemia, melena	Abdominal SPECT/CT scan, 99mTc-pertechnetate scintigraphy Endoscopic resection	Second part of duodenum	None
Cardenas Elias M. A. et al. (5)	2016	Spain	Median age 2.72 y (12 d-7 y)	10	60% M, 40% F	30% presented with acute abdomen, 30% with gastrointestinal bleeding, and 30% were diagnosed prenatally.	Echography, gammagraphy, magnetic resonance, intestinal transit contrast. Laparotomy, partial resection and mucosectomy	Duodenum (10% of cases)	One case of intussusception postsurgical
Cavar S. et al. (6)	2006	Croatia	2 y 10 m; 17 m	2	F F	Obstruction	Ultrasound, barium swallow, CT scan, 99mTc-pertechnetate scintigraphy, macroaggregated albumin scan Laparotomy, marsupialization	Pyloroduodenal duplication; duodenal duplication	None
Born M. Reutter H. (7)	2006	Germany	9y	1	M	Recurrent pain in the right lower abdomen	Laparoscopy	Small intestine	None
de Marliave H. et al. (8)	1985	France	7y	1	F	Severe digestive hemorrhage	Surgery	Small intestine	-

As shown in Table 2, most reported cases present during early childhood, with clinical manifestations largely driven by acid-related mucosal injury, including gastrointestinal bleeding and anaemia (3, 4). In contrast, obstructive presentations are less frequently described and are typically associated with larger or polypoid lesions located in the distal duodenum or duodenojejunal junction (9, 10). Historically, management has been predominantly surgical, although recent reports suggest that endoscopic resection may represent a feasible minimally invasive alternative in selected patients (2, 4).

From a diagnostic perspective, GH remains difficult to identify preoperatively. Endoscopic findings are often non-specific, as lesions may appear as subepithelial or polypoid masses covered by normal mucosa (11). The differential diagnosis of a polypoid duodenal lesion includes adenomatous and hamartomatous polyps, Brunner's gland hyperplasia, enteric duplication, and other subepithelial lesions such as heterotopic pancreas (12, 13). Cross-sectional imaging may identify the lesion and its obstructive effects but lacks specificity (11). In our patient, technetium-99m pertechnetate scintigraphy demonstrated ectopic gastric mucosa, strongly supporting the diagnosis of GH, which was ultimately confirmed by histopathology (4).

Untreated symptomatic GH may lead to complications including ulceration, bleeding, strictures, perforation, intestinal obstruction, and intussusception (7). Accordingly, treatment is aimed at both symptom resolution and prevention of complications (2).

Our case further supports the feasibility of endoscopic management. Nevertheless, complete endoscopic resection may be technically demanding in unfavourably located lesions and may require a staged approach, as observed in our patient. Careful patient selection and multidisciplinary expertise remain essential to achieve complete resection while minimizing procedural risk (10).

This case adds to the limited paediatric literature by describing a rare polypoid GH located at the distal duodenum, presenting with vomiting rather than bleeding or anaemia. It underscores the importance of considering structural causes, including rare entities such as GH, in children with persistent or progressive vomiting patterns.

## Conclusions

Polypoid GH of the distal duodenum is a rare but important cause of recurrent vomiting and partial upper gastrointestinal obstruction in children. This case highlights the value of combining endoscopy, histopathology and technetium-99m pertechnetate scintigraphy to establish the diagnosis. When technically feasible, endoscopic resection represents a safe and effective minimally invasive therapeutic option.

## Data Availability

The original contributions presented in the study are included in the article/Supplementary Material, further inquiries can be directed to the corresponding author.
